# Partial Biotinidase Deficiency Revealed Imbalances in Acylcarnitines Profile at Tandem Mass Spectrometry Newborn Screening

**DOI:** 10.3390/ijerph18041659

**Published:** 2021-02-09

**Authors:** Ilaria Cicalini, Damiana Pieragostino, Cristiano Rizzo, Sara Verrocchio, Daniela Semeraro, Mirco Zucchelli, Silvia Di Michele, Carlo Dionisi-Vici, Liborio Stuppia, Vincenzo De Laurenzi, Ines Bucci, Claudia Rossi

**Affiliations:** 1 Center for Advanced Studies and Technology (CAST), University “G. d’Annunzio” of Chieti-Pescara, 66100 Chieti, Italy; ilaria.cicalini@unich.it (I.C.); damiana.pieragostino@unich.it (D.P.); sara.verrocchio@gmail.com (S.V.); d.semeraro@unich.it (D.S.); m.zucchelli@unich.it (M.Z.); stuppia@unich.it (L.S.); delaurenzi@unich.it (V.D.L.); ibucci@unich.it (I.B.); 2 Department of Medicine and Aging Science, University “G. d’Annunzio” of Chieti-Pescara, 66100 Chieti, Italy; 3 Department of Innovative Technologies in Medicine & Dentistry, University ‘‘G. d’Annunzio’’ of Chieti-Pescara, 66100 Chieti, Italy; 4 Metabolic Diseases Unit, Bambino Gesù Children Hospital and Research Institute, 00165 Rome, Italy; cristiano.rizzo@opbg.net (C.R.); carlo.dionisivici@opbg.net (C.D.-V.); 5 Department of Pediatrics, “Spirito Santo” Hospital, 65100 Pescara, Italy; silvia.dimichele@ausl.pe.it; 6 Department of Psychological, Health and Territory Sciences, School of Medicine and Health Sciences, “G. d’Annunzio” University, 66100 Chieti, Italy

**Keywords:** biotinidase deficiency, newborn screening, inborn errors of metabolism, metabolic profiling, mass spectrometry

## Abstract

Biotinidase (BTD) deficiency is an autosomal recessive inherited neurocutaneous disorder. BTD recycles the vitamin biotin, a coenzyme essential for the function of four biotin-dependent carboxylases, including propionyl-CoA carboxylase, 3-methylcrotonyl-CoA carboxylase, pyruvate carboxylase, and acetyl-CoA carboxylase. Due to deficient activities of the carboxylases, BTD deficiency is also recognized as late-onset multiple carboxylase deficiency and is associated with secondary alterations in the metabolism of amino acids, carbohydrates, and fatty acids. BTD deficiency can be classified as “profound”, with less than 10% of mean normal activity, and as “partial” with 10–30% of mean normal activity. Newborn screening (NBS) of BTD deficiency is performed in most countries and is able to detect both variants. Moreover, mild metabolic alterations related to carboxylase deficiency in profound BTD deficiency could result and possibly be revealed in the metabolic profile by tandem mass spectrometry (MS/MS) NBS. Here, we report the case of a newborn female infant with an initial suspected BTD deficiency at the NBS test, finally confirmed as a partial variant by molecular testing. Although BTD deficiency was partial, interestingly her metabolic profile at birth and during the follow-up tests revealed, for the first time, alterations in specific acylcarnitines as a possible result of the deficient activity of biotin-dependent carboxylases.

## 1. Introduction

**Figure 1:**
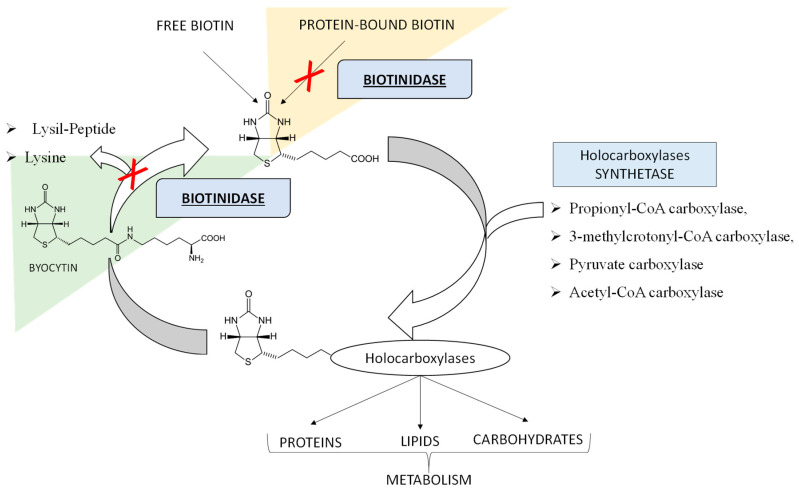
The Biotin Cycle. The figure shows the function of the Biotinidase (BTD) enzyme in making available the waters-soluble vitamin biotin from biocytin and from dietary sources. BTD substrates can be ex novo protein-bound biotin, outside the biotin cycle (as underlined by the orange background) or biocytin resulting from the biotin re-cycle (as underlined by the light green background). In the case of BTD deficiency, as indicated by the red “X”, free biotin, being deficient, cannot properly play its role of coenzyme for the four holocarboxylases, leading to the accumulation of substrates, which causes toxicity and disease signs and symptoms.

Biotinidase (BTD) deficiency is an autosomal recessive inherited disorder of biotin recycling and it is associated with neurologic and cutaneous consequences if untreated [1,2]. The BTD enzyme is encoded by a single gene (BTD) located on chromosome 3p25. Over 150 mutations in the BTD gene have been identified and reported to cause BTD deficiency [1,3,4]. The BTD enzyme, a 70–80 kDa glycoprotein, has a dual function: on the one hand, it separates the water-soluble vitamin biotin from the biocytin, allowing the recirculation of free biotin, also releasing lysine; on the other hand, BTD also recovers biotin from food sources, by breaking down its bond with other proteins [5], as shown in Figure 1.

In particular, biotin in the free form acts as a coenzyme, essential for the function of four carboxylases: propionyl-CoA carboxylase and 3-methylcrotonyl-CoA carboxylase in protein catabolism, pyruvate carboxylase necessary for gluconeogenesis, and acetyl-CoA carboxylase in the first step of fatty acid synthesis [4,6,7]. Therefore, an inherited disorder of biotin recycling because of a deficiency of BTD is also known as late-onset multiple carboxylase deficiency and is associated with secondary imbalances in the metabolism of amino acids, carbohydrates, and fatty acids [8]. Depending on the residual enzymatic activity, BTD deficiency variants are functionally described as “profound” with less than 10% of mean normal activity, and “partial” with 10 to 30% of mean normal activity [1,4,5]. Worldwide, the incidence of BTD deficiency varies from 1:40,000 to 1:60,000 births, being even higher in countries with high consanguinity rates [5]. Interestingly, a higher incidence of 1:6300 (cumulative for complete or partial) has been recently reported in Italy [9]. The initial clinical symptoms in untreated children might appear between two and five months of age, even if they may not be evident until several years later [1]. Untreated infants with profound BTD deficiency can exhibit a variety of neurological and clinical manifestations, including hypotonia, seizures, feeding problems, ataxia, developmental delay, hearing loss, alopecia, eczema, and skin rash [1,4,5]. Biochemically, most untreated patients may present lactic acidosis, ketoacidosis, and eventually hyperammonaemia [1,4]. Other metabolic alterations may include the elevation of lactic acid and alanine due to deficient activity of pyruvate carboxylase, accumulation of propionate, 3-hydroxypropionate, and methyl citrate because of deficient activity of propionyl-CoA carboxylase, and increased excretion levels of 3-hydroxyvaleric acid and 3-methylcrotonylglycine as a result of 3-methylcrotonyl-CoA carboxylase deficiency [1,5,10,11]. Consequently, sometimes in profound BTD deficiency mildly elevated 3-hydroxyvalerylcarnitine (C5OH) and propionylcarnitine (C3) may be revealed [1] by tandem mass spectrometry (MS/MS) analysis as a newborn screening (NBS) test for other inborn errors of metabolism (IEMs) through amino acids and acylcarnitines determination. The over described metabolic alterations as a consequence of multiple carboxylase deficiency have been already reported and confirmed in mice with profound BTD deficiency [12,13,14]. Most patients with partial BTD deficiency are asymptomatic but can develop hypotonia or skin rash under stressful conditions such as fever or starvation. Since symptoms of this metabolic disorder can be prevented and easily treated with success by supplementation with oral biotin in the free form, and an affordable and reliable screening test is available, newborns are screened for BTD deficiency in most countries [1,4]. In Italy, BTD deficiency is one of the disorders incorporated in the nationwide screening program that also includes testing for disorders of amino acid metabolism, urea cycle metabolism, organic acid metabolism, and for defects of fatty acid oxidation, galactosemia, cystic fibrosis, and congenital hypothyroidism. Nowadays, it is well known that metabolic findings and imbalances revealed at NBS may be influenced not only by the genome, but also by other factors such as partial enzyme deficiencies, treatments, nutritional deficiency, prematurity, and maternal defects [15,16,17]. In fact, in screening newborns for BTD deficiency, both profound and partial variants can be detected [1,18]. Sometimes, the profound defects have been associated with metabolic alterations at MS/MS NBS linked to the multiple blocks of carboxylases [1,19].

Here, we describe the case of a newborn female infant who, after the initial detection of BTD deficient activity at NBS, was finally diagnosed with partial BTD deficiency in a compound heterozygous variant. More importantly, we report the first description of alterations in the levels of C3 and C5OH by MS/MS NBS possibly linked to the multiple blocks of carboxylases in a neonatal case of a partial BTD deficiency variant. In fact, even though the deficiency variant identified was partial, MS/MS metabolic profile at birth and during the follow-up tests unexpectedly revealed alterations in specific acylcarnitines, probably related to the deficient function of some of the carboxylases which use biotin as a coenzyme.

## 2. Case Report

### 2.1. Clinical Presentation

The patient, a newborn female infant, was the first child of Caucasian non-consanguineous parents. She was born at 40 weeks of gestation by vaginal delivery, the birth weight was adequate for the gestational age (3600 kg). Neonatal parameters were unremarkable, she presented normal red reflex examination and oto-acoustic emission screening tests. The family history was positive only for maternal thyroid disease. The newborn tested positive at the NBS performed on the second day of life for reduced BTD activity (50.1 U/dL, cut-off > 85 U/dL). The newborn was re-called and the new test confirmed the reduced enzymatic activity. Subsequent follow-up BTD activity determination and BTD gene molecular analysis confirmed a partial BTD deficit. At the initial clinical evaluation, no signs or symptoms consistent with BTD deficiency were observed. Treatment with biotin was recommended only during stress conditions and regular clinical and biochemical follow-ups were scheduled [20]. At subsequent clinical checks, the absence of signs and symptoms and normal psychomotor development were documented. During the COVID-19 pandemic, a family screening was also performed. The father, a 33-year-old, completely asymptomatic adult, also showed a reduced BTD activity associated with an alteration of the acylcarnitine panel, as detailed in the following paragraphs.

### 2.2. Newborn NBS Analysis

**Figure 2:**
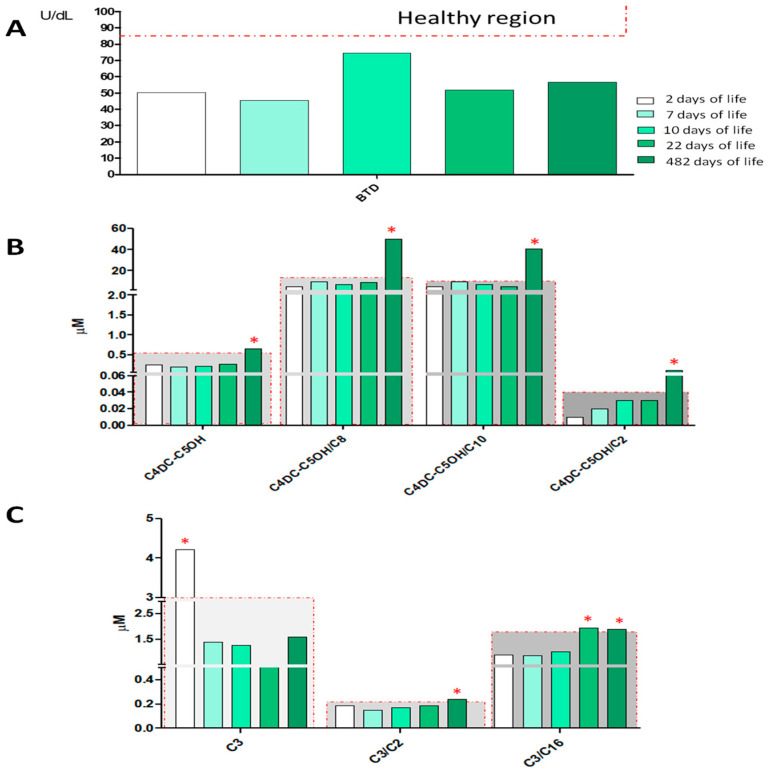
Histograms in the figure show the levels of BTD activity (Panel **A**), C4DC-C5OH (methylmalonyl-3-hyroxy-isovalerylcarnitine) and its ratios C4DC-C5OH/C2, C4DC-C5OH/C10, C4DC-C5OH/C16 (Panel **B**), and the level of C3, C3/C2, C3/C16 (Panel C) of the NBS analyses at 2, 7, 10, 22, and 482 days of life. The samples inside the gray region are considered within the reference limits, while samples falling outside the gray region and marked with red asterisks are outside of the reference limits for newborn screening.

BTD enzyme activity was screened by a fluorescence-based assay (GSP Neonatal Biotinidase, Wallac Oy PerkinElmer) on a dried blood spot (DBS) sample collected at 48 h of life. The newborn screened positive, having BTD activity equal to 50.1 U/dL below the laboratory 85 U/dL cut-off. Based on BTD activity distribution in the laboratory population, the newborn’s value at first DBS was consistent with partial BTD deficiency. The newborn was re-called at 7 and 10 days of life for re-determination of BTD activity. Low BTD activity was confirmed in the repeated DBS: 45.4 U/dL and 74.4 U/dL, respectively, and the newborn was referred to clinical evaluation and genetic analysis. The biochemical phenotype was confirmed at the time of the fourth and fifth DBS collection (Figure 2, Panel A). All DBS samples of the newborn were also analyzed by MS/MS for the simultaneous quantification of amino acids and acylcarnitines. As shown by the histograms in Figure 2B, C several numerous alterations were observed in the acylcarnitine panel. In particular, after 482 days of life of the newborn, the screening of amino acids and acylcarnitines showed an increase in the values of methylmalonyl-3-hyroxy-isovalerylcarnitine C4DC-C5OH = 0.65 μM (cut-off > 0.57 μM), accompanied by an important alteration of its ratio with short and medium-chain acylcarnitines: C4DC-C5OH/C2 = 0.10 (cut-off > 0.04), C4DC-C5OH/C8 = 50.2 (cut-off > 16), and C4DC-C5OH/C10 = 40.75 (cut-off > 11), as reported in Figure 2, Panel B. While screening analyses in MS/MS showed an alteration of proprionylcarnitine (C3), already in the first sample at 2 days of life (Figure 2, Panel C). The alteration of acylcarnitine C3 (sampling after 2 days of life) and the alteration of the ratios C3/C2 and C3/C16 (after 482 days of life) required a further second-level analysis for the quantification of homocysteine (HCY), acid methyl citric acid (MCA) and methylmalonic acid (MMA), showing, in both cases, values below the reference limit for all the three monitored metabolites. Table 1 shows in detail all the information relating to the first and second-level analyses of all the samples collected from the newborn. Methodological details are reported in the supplementary materials and in Table S1.

**Figure 3:**
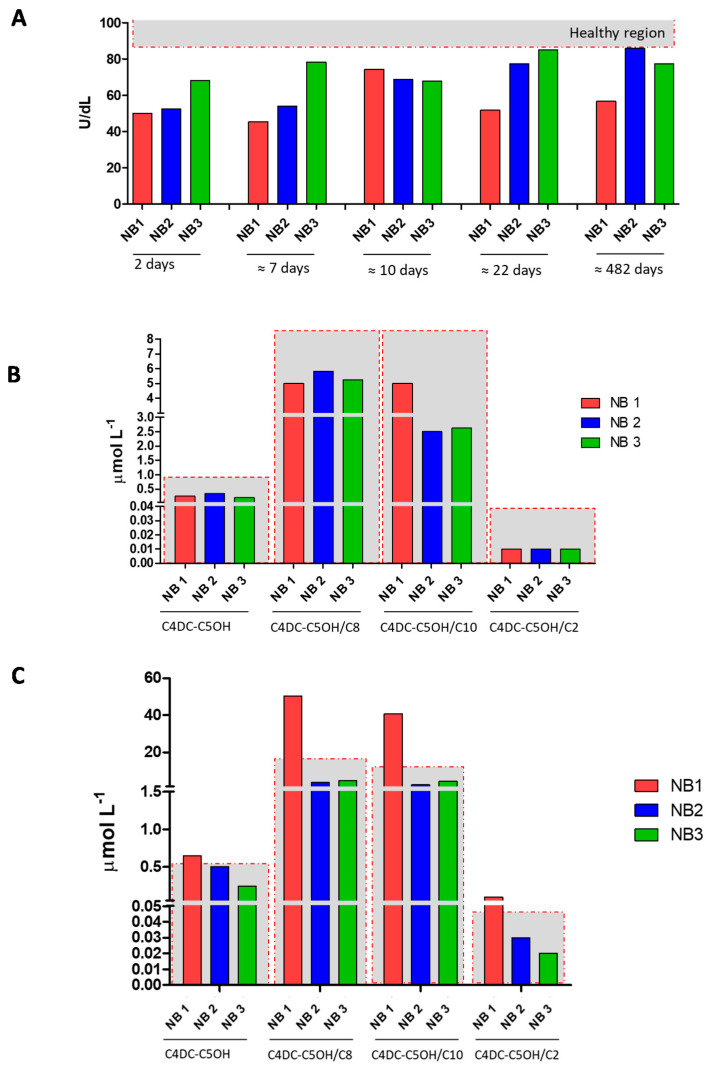
Panel **A** shows BTD activity measured in dried blood spot (DBS) samples from the newborn subject of the case report (NB1 in red) and the other two newborns with partial BTD deficiency (NB2 and NB3 in blue and green, respectively) at 2, 7, 10, 22, and 482 days of life. Panel **B** shows the levels of C4DC-C5OH and its ratios C4DC-C5OH/C2, C4DC-C5OH/C8, and C4DC-C5OH/C10 for NB1, NB2, and NB3 at 2 days of life. Panel **C** shows the levels of C4DC-C5OH and its ratios C4DC-C5OH/C2, C4DC-C5OH/C8, and C4DC-C5OH/C10 for NB1, NB2, and NB3 at about 482 days of life. The samples inside the gray region are considered within the reference limits, while samples falling outside the gray region and marked with red asterisks are outside of the reference limits for newborn screening.

In order to emphasize the metabolic alterations observed in the present neonatal case of partial BTD deficiency, we show in Figure 3 the comparison with the other two newborns with a partial defect of BTD, at different time points (Figure 3, Panel A). Interestingly, histograms in Panel C highlight no alteration in the levels of C4DC-C5OH and its ratios for the two newborns (NB2 and NB3) involved in the comparison. In addition, in Figure S1 we also report the comparison between the newborn discussed in the present case report and a few of the negative neonatal samples in terms of BTD activity and levels of acylcarnitine C4DC-C5OH and its ratios, as examples of normal ranges in the neonatal population.

### 2.3. Parents’ NBS Analysis

**Figure 4:**
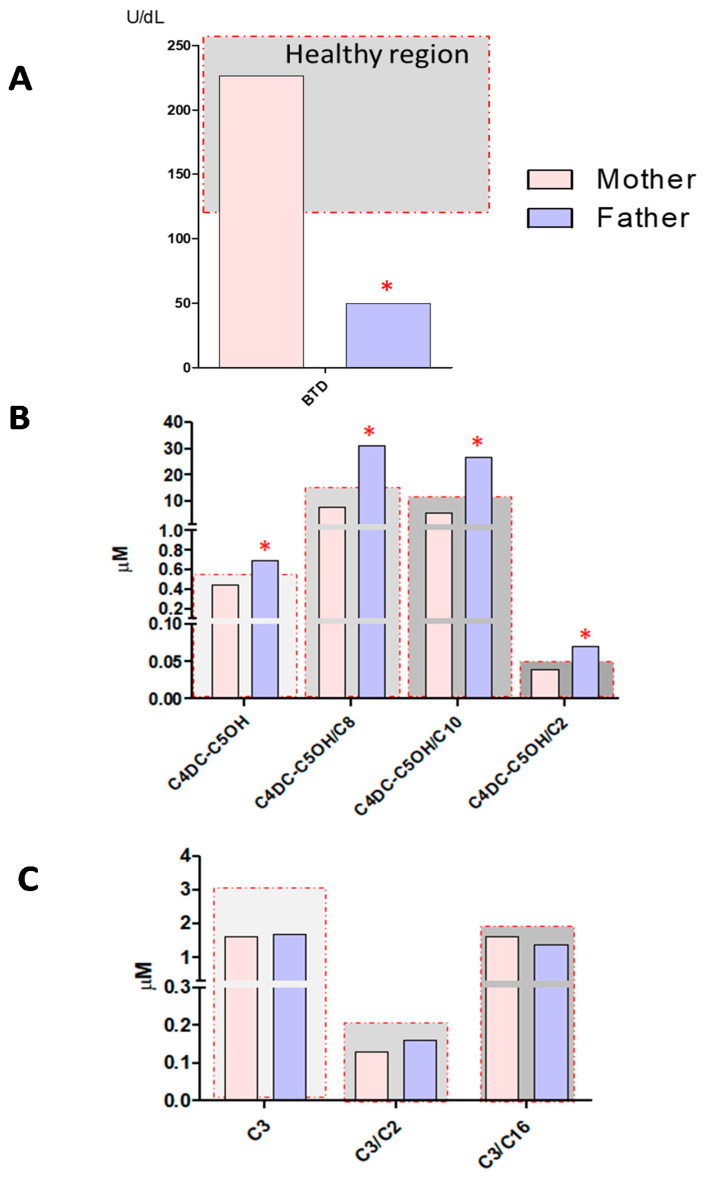
Panel **A** shows BTD activity measured in the DBS samples from the mother and father of the newborn. Panel **B** shows the levels of C4DC-C5OH and its ratios C4DC-C5OH/C2, C4DC-C5OH/C8, and C4DC-C5OH/C10 for DBS samples from the mother and father of the newborn. Panel **C** shows levels of C3 and its ratios C3/C2, C3/C16 in the DBS samples from the mother and father of the newborn. The samples inside the gray region are considered within the reference limits, while samples falling outside the gray region and marked with red asterisks are outside of the reference limits for newborn screening.

The BTD deficiency DBS screening test was also performed on the newborn’s parents. As shown in Figure 4, Panel A, the father, a 33-year-old adult, completely asymptomatic, showed a reduced BTD activity of 49.9 U/dL, (cut-off > 85 U/dL), while the BTD activity measured in the mother’s sample was found to be fully normal. Interestingly, at MS/MS analyses, the father’s sample showed an evident increase in the levels of acylcarnitine C4DC-C5OH = 0.70 μM (cut-off > 0.57 μM) and of its ratio with short and medium-chain acylcarnitines: C4DC-C5OH/C2 = 0.07 (cut-off > 0.04), C4DC-C5OH/C8 = 31.0 (cut-off > 16), and C4DC-C5OH/C10 = 26.74 (cut-off > 11), as reported in Figure 4, Panel B. On the other hand, no alterations in the levels of acylcarnitine C3 and in its ratios were found (Figure 4, Panel C). The mother’s MS/MS profile was unremarkable.

### 2.4. BTD Gene Molecular Analysis and Diagnostic Confirmation

Genomic DNA from peripheral blood and sequence analysis of the BTD gene was performed by Next Generation Sequencing (NGS) (NovaSeq6000, Illumina, San Diego, CA, USA) using NimbleGen SeqCap Target Enrichment (Roche, Basel, Switzerland). According to the sequence analysis results of the BTD gene, including exon-intron boundaries, the BTD deficiency was finally confirmed as compound heterozygous. The mutations found were p.(Val62Met) and p.(Asp444His) corresponding to the following genotypes: c.184G > A and c.1330G > C. To date, the segregation has not yet been confirmed in the parents. Molecular testing results together with the percentage of residual enzymatic activity measured by fluorescence-based BTD assays at NBS allowed us to confirm the partial BTD deficiency variant.

## 3. Discussion

BTD deficiency screening allows the estimation of enzyme activity and identification of newborns requiring diagnostic confirmation. Newborns affected with profound BTD deficiency have less than 10% of mean normal enzyme activity, while 10–30% of mean normal enzyme activity is observed in the partial form [4]. In this case, we report the story of a female full-term infant from non-consanguineous parents, in which suspected BTD deficiency at NBS was confirmed as a partial form at the genetic analysis. As already described and reported in Figure 1, this enzyme recycles the vitamin biotin, a coenzyme essential for the function of four biotin-dependent carboxylases, including propionyl-CoA carboxylase, 3-methylcrotonyl-CoA carboxylase, pyruvate carboxylase, and acetyl-CoA carboxylase [1,4]. In Italy, the expanded NBS program includes, in addition to the measurement of BTD activity through fluorescence-based assays, the measurement of total galactose and a large panel of amino acids and acylcarnitines in DBS samples thanks to the use of high-throughput technologies such as MS/MS [21]. Importantly, the entire metabolites panel allows appreciating possible alterations related to propionyl-CoA carboxylase and 3-methylcrotonyl-CoA carboxylase activity, as biotine-depended enzymes. In particular, mildly elevated C3 levels may reflect propionyl-CoA carboxylase deficiency. As well, increased levels of C5OH may be linked to 3-methylcrotonyl-CoA carboxylase activity. Rai-Hseng Hsu et al. have already described cases of neonates diagnosed with BTD deficiency, who simultaneously presented alterations of C5OH and elevation of urinary levels of 3-hydroxyisovalerate, 3-methylcrotonylglycine, lactate, and pyruvate [19]. However, these described cases are always related to a profound deficit in BTD activity. Here, we report, for the first time, some slight alterations in the levels of C3 and C5OH at MS/MS NBS in a neonatal case of partial BTD deficiency. The MS/MS analyses carried out in our laboratory showed an alteration of the C3, already in the first sample (2 days of life). Then, during the last follow-up (after 482 days of life), increased ratios of C3/C2 and C3/C16 were detected. These metabolic alterations at MS/MS NBS required second-tier tests by LC-MS/MS for the quantification of methyl citric acid (MCA), as a direct product of propionyl-CoA carboxylase activity. MCA levels, normally undetectable, were quantified only in the last LC-MS/MS analysis after 482 days of life (MCA = 0.8 μM, cut-off < 1 μM), highlighting a slight accumulation of MCA, probably linked to a deficiency in the disposal of propionyl-CoA through propionyl-CoA carboxylase activity [22]. Furthermore, the levels of C4DC-C5OH and its ratios with short and medium-chain acylcarnitines were found to be within the reference limits in the first four samples between 2 and 22 days of life. Instead, it is interesting to note that NBS analyses carried out on the last sampling (after 482 days of life) revealed alterations of C4DC-C5OH and its ratios. These data might be suggestive of deficient 3-methylcrotonyl-CoA carboxylase activity, considering the strong inverse correlation between C5OH levels and residual enzymatic activity [23,24]. Surprisingly, her father, who showed approximately the same residual BTD activity, also presented similar alterations in his metabolic profile. More specifically, C4DC-C5OH and its ratios versus C2, C8, and C10 were found to be increased after MS/MS analysis of the father’s DBS sample.

## 4. Conclusions

The present neonatal case highlights the importance of an expanded NBS test in detecting partial enzyme deficiencies, also allowing for subclassifications of the disorders. Moreover, the expanded NBS approach also allows revealing patients with late-onset forms of diseases, which may never require treatment or only in adulthood. As already discussed, BTD deficiency could be secondarily accompanied by acidosis and a characteristic organic acidemia due to multiple carboxylase deficiency [9]. For this reason, even in countries where an NBS test for BTD deficiency is not performed, we highly recommend that newborns with specific abnormalities in acylcarnitine profile following MS/MS NBS be considered for suspicion of BTD deficiency. In addition, plasma acylcarnitines by Liquid Chromatography coupled to tandem Mass Spectrometry (LC-MS/MS) and urinary organic acid profiles by Gas Chromatography-Mass Spectrometry (GC-MS) should be performed to check for the altered results at the NBS test. Finally, the present case points out the significance of the follow-up, mainly in asymptomatic patients. In fact, follow-up analyses of the patient allow appreciating any further alterations as indices of the metabolic status.

## Figures and Tables

**Table 1:** Results of newborn screening (NBS) and second-level tests carried out at 2, 7, 10, 22, and 482 days of life. Results in bold and italics characters and with an asterisk indicate positive test results. N.d. means: Not Detected.

Analyte	Time of Collection					Normal Value
	2 Days of Life	7 Days of Life	10 Days of Life	22 Days of Life	482 Days of Life	
**BTD (U/dL)**	50.10 *	45.4 *	74.4 *	51.8 *	56.60 *	>85
**C4DC-C5OH (µM)**	0.25	0.20	0.21	0.27	0.65*	<0.57
**C4DC-C5OH/C2**	0.01	0.02	0.03	0.03	0.10*	<0.04
**C4DC-C5OH/C8**	5.0	10.0	7.0	9.0	50.20*	<16
**C4DC-C5OH/C10**	5.0	10.0	7.0	5.4	40.75*	<11
**C3 (µM)**	4.22*	1.39	1.26	0.51	1.60	<3.3
**C3/C2**	0.19	0.15	0.17	0.19	0.24 *	<0.20
**C3/C16**	0.88	0.85	1.02	1.96 *	1.91 *	<1.50
**HCY (µM)**	4.7	---	---	---	4.3	<10
**MMA (µM)**	0.7	---	---	---	0.7	<4
**MCA (µM)**	n.d.	---	---	---	0.8	<1

## Supplementary Materials

The following are available online at https://www.mdpi.com/1660-4601/18/4/1659/s1, Table S1, Figure S1.
