# Supplementary material for: Partial Biotinidase Deficiency Revealed Imbalances in Acylcarnitines Profile at Tandem Mass Spectrometry Newborn Screening
Source: Int J Environ Res Public Health. 2021 Feb 9;18(4):1659. doi: 10.3390/ijerph18041659 (PMC7916230; doi:10.3390/ijerph18041659)
Supplement: Supplementary file 1 [file ijerph-18-01659-s001.pdf]

Supplementary Material of:

Partial biotinidase deficiency revealed imbalances in acylcarnitines profile at tandem mass spectrometry newborn screening

Ilaria Cicalini [1,2], Damiana Pieragostino [1,3], Cristiano Rizzo [4], Sara Verrocchio [1,2], Daniela Semeraro [1,2], Mirco Zucchelli [1,3], Silvia Di Michele [5], Carlo Dionisi-Vici [4], Liborio Stuppia [1,6], Vincenzo De Laurenzi [1,3], Ines Bucci [1,2] and Claudia Rossi [1,6,*]

1 Center for Advanced Studies and Technology (CAST), University "G. d'Annunzio" of Chieti-Pescara, 66100 Chieti, Italy; ilaria.cicalini@unich.it (I.C.); damiana.pieragostino@unich.it (D.P.); sara.verrocchio@gmail.com (S.V.); d.semeraro@unich.it (D.S.); m.zucchelli@unich.it (M.Z.); stuppia@unich.it (L.S.); delaurenzi@unich.it (V.D.L.); ibucci@unich.it (I.B.)
2 Department of Medicine and Aging Science, University "G. d'Annunzio" of Chieti-Pescara, 66100 Chieti, Italy
3 Department of Innovative Technologies in Medicine & Dentistry, University ''G. d'Annunzio'' of Chieti-Pescara, 66100 Chieti, Italy
4 Metabolic Diseases Unit, Bambino Gesù Children Hospital and Research Institute, 00165 Rome, Italy; cristiano.rizzo@opbg.net (C.R.); carlo.dionisivici@opbg.net (C.D.-V.)
5 Department of Pediatrics, "Spirito Santo" Hospital, 65100 Pescara, Italy; silvia.dimichele@ausl.pe.it
6 Department of Psychological, Health and Territory Sciences, School of Medicine and Health Sciences, "G. d'Annunzio" University, 66100 Chieti, Italy
* Correspondence: claudia.rossi@unich.it; Tel.: +39-0871-541596; Fax: +39-0871-541598

Routinely Newborn Screening Analysis

Dried blood spot (DBS) samples for NBS are punched out into 3.2 mm-disks to perform a flow injection-tandem mass spectrometry analysis (FIA-MS/MS) for the detection of 36 IEMs, including AAs, urea cycle, organic acid and fatty acid oxidation disorders. Actually, four 3.2 mm DBS disks are used for immunofluorimetric assays by Genetic Screening Processor GSP® Instrument (Perkin Elmer Life and Analytical Sciences, Turku, Finland) to test congenital hypothyroidism (CH), cystic fibrosis (CF), galactosemia and biotinidase deficiency, respectively, and the fifth DBS disk is employed for FIA-MS/MS analysis. For the latter, the DBS disk (of approximately 3-3.2 µL whole blood) is extracted for the determination of 14 AAs, 35 acylcarnitines (ACs), free carnitine and succinylacetone, by using the NeoBase 2 Non-Derivatized MSMS Kit (Perkin Elmer Life and Analytical Sciences, Turku, Finland). The MS/MS system consists RenataDX™ Screening Systems Waters Corporation, Milford, MA, USA) as a fully FIA-MS/MS IVD system for high-throughput analysis. The system components include the 3777C IVD Sample Manager, ACQUITY™ UPLC™ I-Class IVD Binary Solvent Manager, and Xevo™ TQD IVD Mass Spectrometer (Waters Corporation, Milford, MA, USA). The system operates in positive electrospray ionization mode by multiple reaction monitoring (MRM) acquisition. 10 µL are injected into the ion source and the run time is 1.3 min, injection-to-injection. Data are finally processed by MassLynx™ (IVD) Software V4.2 and IonLynx™ Application Manager (Waters Corp.). MS parameters are listed in Supplementary Table S1, considering MS/MS transitions, cone potentials and collision energies for each metabolites and their internal standards.

Table S1:

Analytes and ISs	Transition	Cone potential	Collision energy
Ala<br>**$^{2}H_{3}$-Ala**	90.1>44.0<br>93.1>47.1	22	8
Arg<br>**$^{2}H_{4}$, ^{13}C-Arg**	175.1>70.1<br>180.1>75.1	34	21

Asa<br>**$^{2}H_{4}$, ^{13}C-Arg**	291.1>70.1<br>180.1>75.1	42<br>34	30<br>21
Cit<br>**2H$_{2}$-Cit**	176.1>113.1<br>178.1>115.1	24	16
Gly<br>**$^{15}N_{2}$-^{13}C-Gly**	76.0>30.0<br>78.0>32.0	22	7
Leu/Ile/Pro-OH<br>**$^{2}H_{3}$-Leu**	132.1>86.1<br>135.1>89.1	24	10
Met<br>**$^{2}H_{3}$-Met**	150.1>104.1<br>153.1>107.1	24	10
Orn<br>**$^{2}H_{6}$-Orn**	133.1>70.1<br>139.1>76.1	22	16
Phe<br>**$^{13}C_{6}$-Phe**	166.1>120.1<br>172.1>126.1	25	12
Tyr<br>**$^{13}C_{6}$-Tyr**	182.1>136.1<br>188.1>142.1	26	12
Val<br>**$^{15}N_{2}$-$^{13}C_{5}$-Val**	118.1>72.1<br>124.1>77.1	23	10
Pro<br>**$^{13}C_{5}$-Pro**	116.1>70.1<br>121.1>74.1	28	12
Gln/Lys<br>**$^{13}C_{5}$-Gln**	147.1>84.0<br>152.1>88.1	22	16
Glu<br>**$^{13}C_{5}$-Gln**	148.1>84.0<br>152.1>88.1	24<br>22	14<br>16
C0<br>**$^{2}H_{9}$-C0**	162.1>1030<br>171.2>103.0	38	16
C2<br>**$^{2}H_{3}$-C0**	204.1>85.0<br>207.1>85.0	34	18
C3<br>**$^{2}H_{3}$-C3**	218.1>85.0<br>221.2>85.0	32	18
C4<br>C3DC/C4OH<br>**$^{2}H_{3}$-C4**	232.2>85.0<br>248.1>85.0<br>235.2>85.0	36	18
C5<br>C5:1<br>C4DC/C5OH<br>**$^{2}H_{9}$-C5**	246.2>85.0<br>244.2>85.0<br>262.1>85.0<br>255.2>85.0	38	20
C6<br>**$^{2}H_{3}$-C6**	260.2>85.0<br>263.2>85.0	37	20
C5DC/C6OH<br>C6DC<br>**$^{2}H_{6}$-C5DC**	276.2>85.0<br>290.2>85.0<br>282.2>85.0	40	24
C8:1<br>C8<br>**$^{2}H_{3}$-C8**	286.2>85.0<br>288.2>85.0<br>291.2>85.0	42	22
C10<br>C10:2<br>C10:1<br>**$^{2}H_{3}$-C10**	316.2>85.0<br>312.2>85.0<br>314.2>85.0<br>319.3>85.0	45	22
C12<br>C12:1<br>**$^{2}H_{3}$-C12**	344.3>85.0<br>342.3>85.0<br>347.3>85.0	46	24
C14<br>C14:1	372.3>85.0<br>370.3>85.0	52	25

C14:2<br>C14OH<br>**$^{2}H_{3}$-C14**	368.3>85.0<br>388.3>85.0<br>375.3>85.0		
C16:1<br>C16<br>C16OH<br>**$^{2}H_{3}$-C16**	398.3>85.0<br>400.3>85.0<br>416.3>85.0<br>403.4>85.0	55	26
C16:1OH/C17<br>**$^{2}H_{3}$-C16**	414.3>85.0<br>403.4>85.0	55	26
C18<br>C18:1<br>C18:2<br>C18:1OH<br>C18OH<br>C18:2OH<br>**$^{2}H_{3}$-C18**	428.4>85.0<br>426.4>85.0<br>424.3>85.0<br>442.4>85.0<br>444.4>85.0<br>440.3>85.0<br>431.4>85.2	56	28
C20<br>C22<br>C24<br>C26<br>**$^{2}H_{3}$-C26**	456.4>85.0<br>484.4>85.0<br>512.5>85.0<br>540.5>85.0<br>543.5>85.0	69	34
SA<br>**$^{13}C_{5}$-MPP**[2]	155.1>109.1<br>160.1>114.1	24	22

Table S1. Table listed the MS/MS parameters (cone voltage (V) and collision energy (eV)) and MRM transition used for the quantification of metabolites. The internal standards (ISs) are reported in bold.

Routinely Newborn Screening Analysis for Biotinidase deficiency

For biotinidase activity test, the DBS sample is punched into final diameter disks of approximately 3.2 mm, using an automatic Puncher (Panthera-Puncher, PerkinElmer). Enzyme activity is determined using a quantitative kit, the GSP Neonatal Biotinidase kit (PerkinElmer) and a Genetic Screening Processor (GSP instrument, PerkinElmer). This assay combines an enzyme reaction with a solid phase time-resolved immunofluorescence assay. In particular, europium-labeled biotin is used as artificial substrate in the fluorescence-based assay, and the europium fluorescence is inversely proportional to the biotinidase activity of the sample. Finally, the biotinidase enzyme cleave the amide bond in europium-labeled biotin, after the enzyme reaction is stopped by addition of streptavidin which has high affinity for biotin. Results of screening are processed and stored in the GSP Workstation software.

Second-tier test for the quantification of methylmalonic acid, methylcitric acid and homocysteine by LC-MS/MS

For C3 second tier test, the DBS sample is punched out twice into final diameter disks of approximately 3.2 mm, using an automatic puncher, following the method already described in our previous work [1]. Briefly, two DBS disks (approximately 6-6.4 µL whole blood) are extracted for the determination of mma (CDN Isotopes), mca (CDN Isotopes) and hcy (Sigma-Aldrich) by LC-MS/MS. Their relative Internal standards $^{2}H_{4}$-hcy and $^{2}H_{3}$-mma were provided from Cambridge Isotope Laboratories, Inc and $^{2}H_{3}$-mca from CDN Isotopes. For hcy, mma and mca quantification by LC-MS/MS analysis, 250 µL of extraction solution, containing ISs 1uM, 1 M Dithiothreitol (DTT, purchased from

Sigma-Aldrich) and acetonitrile/water 70:30 with 0.5% formic acid, were added to two 3.2 mm-DBS disks of sample, calibrators and QCs. Each sample was gently mixed (20°C, 60 min) in a Thermomixer (Eppendorf®). The supernatant was transferred into a new 1.5 mL tube and dried in a SpeedVac. The residue was then reconstituted with 100 µL of 3N HCl in n-Butanol (purchased from Sigma-Aldrich) and mixed in a Thermomixer (65°C, 15 min). The sample was dried once again in a SpeedVac, then the residue was reconstituted with 100 µL of water, briefly centrifuged and the supernatant was transferred into polypropylene vial (provided by Waters Corporation).

The LC-MS/MS system consists of an ACQUITY UPLC I-Class/Xevo TQD IVD tandem quadrupole mass spectrometer (Waters Corporation, Milford, MA, USA), operating in positive ion mode. The chromatographic separation of analytes was performed using ACQUITY UPLC BEH C18 2.1 x 50mm column with ACQUITY UPLC BEG C18 VanGuard pre-column. In a total run time of 9 minutes, the elution of hcy, mma and mca was achieved through a gradient of mobile phases, starting from 5% of to 90% of ACN with Formic acid 0.1% (solvent B), water with Formic acid 0.1% was used as solvent A. The flow rate was 0.5 mL/min.

Figure S1: Panel A shows BTD activity measured in DBS samples from the newborn subject of the case report (NB 1 in red) and other five newborn as an example of negative newborn population (NB 2,NB 3, NB 4, NB 5, NB 6 in green). Panel B shows the levels of C4DC-C5OH and its ratios C4DC-C5OH/C2, C4DC-C5OH/C8, C4DC-C5OH/C10 for NB 1 (newborn subject of the case report, in red) and negative NB 2,NB 3, NB 4, NB 5, NB 6 (in green). The samples inside the gray region are considered within the reference limits, while samples falling outside the gray region and marked with red asterisks are outside of the reference limits for newborn screening**.**

A

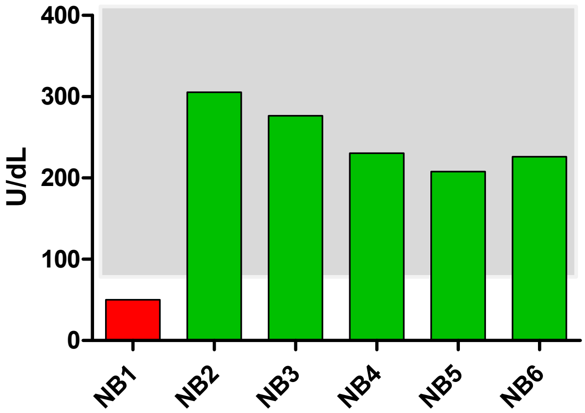

B

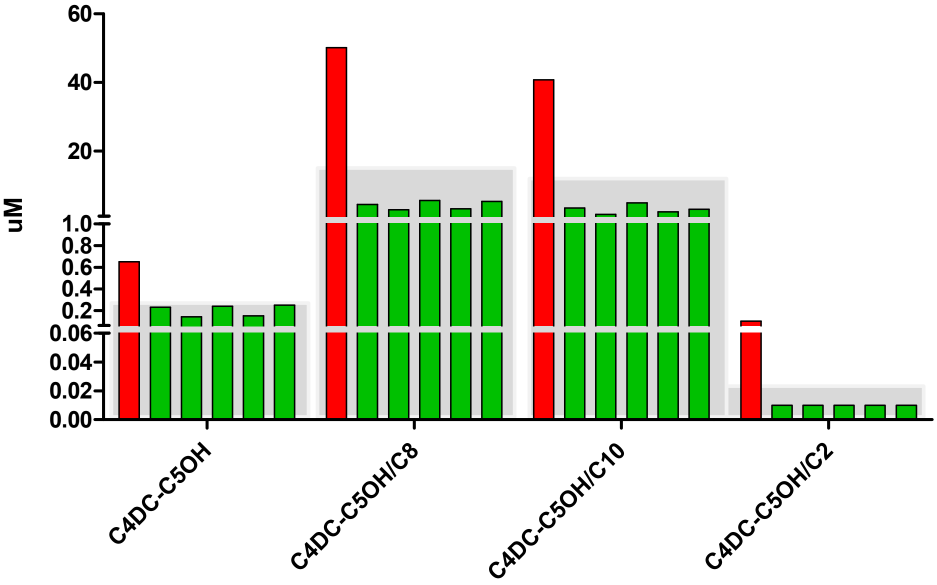

Reference

1. Rossi, C.; Cicalini, I.; Rizzo, C.; Zucchelli, M.; Consalvo, A.; Valentinuzzi, S.; Semeraro, D.; Gasparroni, G.; Brindisino, P.; Gazzolo, D.; Dionisi-Vici, C.; De Laurenzi, V.; Pieragostino, D., A False-Positive Case of Methylmalonic Aciduria by Tandem Mass Spectrometry Newborn Screening Dependent on Maternal Malnutrition in Pregnancy. *International journal of environmental research and public health* **2020,** 17, (10).
